# Research on the Influence of Core Sensing Components on the Performance of Galvanic Dissolved Oxygen Sensors

**DOI:** 10.3390/s24134155

**Published:** 2024-06-26

**Authors:** Helai Liu, Lingfeng Zhang, Ye Wu, Weimin Ding, Yutao Liu, Sanqin Zhao, Jiabing Gu

**Affiliations:** College of Engineering, Nanjing Agricultural University, Nanjing 210031, China; 2021819066@stu.njau.edu.cn (H.L.); 2022812059@stu.njau.edu.cn (L.Z.); 2022812064@stu.njau.edu.cn (Y.W.); wmding@njau.edu.cn (W.D.); ytliu@njau.edu.cn (Y.L.); zhaosanqin@njau.edu.cn (S.Z.)

**Keywords:** galvanic cell type, dissolved oxygen sensor, oxygen-permeable membrane, electrode, electrolyte, performance analysis

## Abstract

The galvanic dissolved oxygen sensor finds widespread applications in multiple critical fields due to its high precision and excellent stability. As its core sensing components, the oxygen-permeable membrane, electrode, and electrolyte significantly impact the sensor’s performance. To systematically investigate the comprehensive effects of these core sensing components on the performance of galvanic dissolved oxygen sensors, this study selected six types of oxygen-permeable membranes made from two materials (Perfluoroalkoxy Polymer (PFA) and Fluorinated Ethylene Propylene Copolymer (FEP)) with three thicknesses (0.015 mm, 0.03 mm, and 0.05 mm). Additionally, five concentrations of KCl electrolyte were configured, and four different proportions of lead–tin alloy electrodes were chosen. Single-factor and crossover experiments were conducted using the OxyGuard dissolved oxygen sensor as the experimental platform. The experimental results indicate that under the same membrane thickness conditions, PFA membranes provide a higher output voltage compared to FEP membranes. Moreover, the oxygen permeability of FEP membranes is more significantly affected by temperature. Furthermore, the oxygen permeability of the membrane is inversely proportional to its thickness; the thinner the membrane, the better the oxygen permeability, resulting in a corresponding increase in sensor output voltage. When the membrane thickness is reduced from 0.05 mm to 0.015 mm, the sensor output voltage for PFA and FEP membranes increases by 86% and 74.91%, respectively. However, this study also observed that excessively thin membranes might compromise measurement accuracy. In a saturated, dissolved oxygen environment, the sensor output voltage corresponding to the six oxygen-permeable membranes used in the experiment exhibits a highly linear inverse relationship with temperature (correlation coefficient ≥ 98%). Meanwhile, the lead–tin ratio of the electrode and electrolyte concentration have a relatively minor impact on the sensor output voltage, demonstrating good stability at different temperatures (coefficient of variation ≤ 0.78%). In terms of response time, it is directly proportional to the thickness of the oxygen-permeable membrane, especially for PFA membranes. When the thickness increases from 0.015 mm to 0.05 mm, the response time extends by up to 2033.33%. In contrast, the electrode material and electrolyte concentration have a less significant effect on response time. To further validate the practical value of the experimental results, the best-performing combination of core sensing components from the experiments was selected to construct a new dissolved oxygen sensor. A performance comparison test was conducted between this new sensor and the OxyGuard dissolved oxygen sensor. The results showed that both sensors had the same response time (49 s). However, in an anaerobic environment, the OxyGuard sensor demonstrated slightly higher accuracy by 2.44%. This study not only provides a deep analysis of the combined effects of oxygen-permeable membranes, electrodes, and electrolytes on the performance of galvanic dissolved oxygen sensors but also offers scientific evidence and practical guidance for optimizing sensor design.

## 1. Introduction

With the increasing importance of environmental protection and water quality monitoring, dissolved oxygen (DO) serves as a critical indicator of water body health and the ecological environment [1,2]. Its accurate measurement is indispensable in fields such as environmental protection [3], aquatic ecology research [4], and sewage treatment [5]. The galvanic DO sensor has gradually become one of the mainstream technologies for DO detection [6,7] due to its good stability [8], rapid response [9], and convenient use [10].

**Figure 1:**
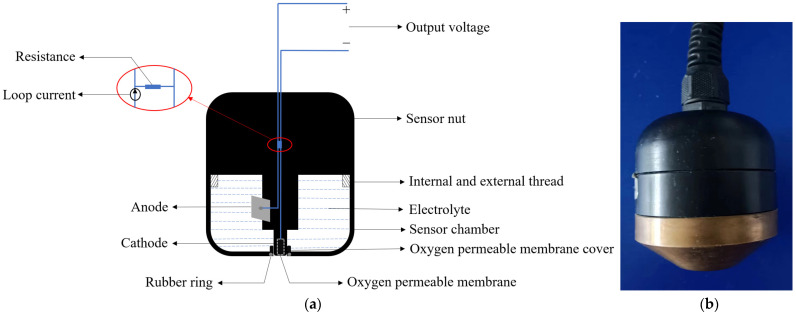
Galvanic DO sensor. (**a**) Structural diagram; (**b**) physical image.

The overall structure of the galvanic DO sensor is shown in Figure 1a [11]. It mainly consists of multiple components precisely assembled, including a gold-plated cathode, a lead alloy anode, an oxygen-permeable membrane, electrolyte, conductive lines, a membrane cover, and a sealing rubber ring [12,13]. Its working principle is based on electrochemical reactions, where the concentration of DO is determined by measuring the current generated by the redox reaction between the DO and the electrodes [14]. During the measurement process, the sensor is placed in the water sample to be tested. The DO in the water sample penetrates through the oxygen-permeable membrane into the electrolyte, triggering a redox reaction on the cathode and anode. The reaction equations are shown in Equations (1) to (3) [15]. Oxygen molecules react at the cathode of the sensor and are consumed, causing an imbalance in DO concentration inside and outside the oxygen-permeable membrane. Oxygen molecules in the tested water sample diffuse through the membrane to the cathode, where they are reduced to OH^−^ and form a closed circuit with the external circuit, generating a loop current. The magnitude of the loop current is directly proportional to the DO concentration and is converted into a voltage analog output through a resistor, thus enabling the measurement of DO [16]. The physical appearance of the galvanic-cell-type dissolved oxygen sensor is shown in Figure 1b.

O_2_ + 2H_2_O + 4e^−^ → 4OH^−^ (1)

2Pb → 2Pb^2 +^ + 4e^−^ (2)

2Pb + O_2_ + 2H_2_O → 2Pb(OH)_2_ (3)

The performance of DO sensors has a direct impact on the accuracy and reliability of measurement results. Therefore, it is particularly important to conduct in-depth exploration of the key factors that affect sensor performance. Among the many influencing factors, the roles of the oxygen-permeable membrane, electrodes, and electrolyte cannot be ignored. The oxygen-permeable membrane [17], as the sensing part of the DO sensor, bears the function of oxygen molecule permeation and sensing. Its material and thickness not only determine the passage rate of oxygen molecules but also directly relate to the output intensity of the sensor signal. Fluoroplastics such as PFA [18] and FEP [19] are widely used in the manufacture of oxygen-permeable membranes due to their excellent chemical stability and oxygen permeability. The electrode is another core component of the sensor, and its material and composition are directly related to the efficiency and stability of the electrochemical reaction. Among them, lead–tin alloy is often chosen as the electrode material because of its excellent conductivity and corrosion resistance [20]. In addition, the electrolyte, as the medium for electrochemical reactions, provides the necessary ionic conductive environment for the electrochemical reaction of oxygen. Potassium chloride (KCl) is one of the most commonly used electrolytes, and its concentration directly affects the output voltage of the sensor [21]. The oxygen-permeable membrane, electrodes, and electrolyte are key factors that affect sensor performance, and in-depth research and optimization of them will help improve the overall performance of the sensor.

Previous research on galvanic DO sensors has mainly focused on optimizing their performance by improving different materials [22] or processes [23,24]. For example, Hossain et al. [25] used a carbon dioxide laser to pattern laser-induced graphene on a polyimide membrane and subsequently electrodeposited colloidal platinum to form an electroactive sensing area, achieving selective detection of DO. Sosna et al. [26] designed a bare microdisk electrode suitable for seawater detection. This method eliminates the need for frequent replacement of the oxygen-permeable membrane, enabling direct measurement of DO concentration and exhibiting excellent linearity and stable current response. In addition, Luo et al. [27] selected polydimethylsiloxane as the material for the oxygen-permeable membrane, effectively reducing the sensor’s response time. However, few studies have comprehensively considered the combined effects of the oxygen-permeable membrane, electrodes, and electrolyte on DO sensor performance at this stage. This study aims to deeply explore and analyze these factors by selecting oxygen-permeable membranes of different materials and thicknesses, various proportions of lead–tin alloy electrodes, and different concentrations of KCl electrolyte. The following are the main contributions of this paper:

1. Through single-factor experiments, the critical influence of the oxygen-permeable membrane on the performance of the DO sensor is deeply analyzed.
2. With the help of crossover experiments, the synergistic effects of electrodes and electrolyte on DO sensors are comprehensively explored.
3. From the perspective of core sensing components, it provides detailed data support and practical suggestions for optimizing sensor design.

Next, the arrangement of each section of this article will be explained: Section 2 will introduce the materials and equipment used in the research, experimental methods, and specific experimental processes in detail. Section 3 focuses on exploring how oxygen permeation efficiency specifically affects the output signal of the DO sensor and summarizes and discusses the experimental results on the stability and response time of the sensing component’s output signal in the DO sensor. Finally, a general summary of the full text will be given in the fourth section.

## 2. Materials and Methods

### 2.1. Experimental Materials

Two different types of oxygen-permeable membranes were used in the experiment: PFA film and FEP film. For each type of oxygen-permeable membrane, three thicknesses were selected: 0.015 mm, 0.03 mm, and 0.05 mm. For ease of data recording, they were labeled as 0.015 mm PFA, 0.03 mm PFA, 0.05 mm PFA, 0.015 mm FEP, 0.03 mm FEP, and 0.05 mm FEP. All these oxygen-permeable membranes were produced by Guangzhou Polyfluor New Material Technology Co., Ltd. (Guangzhou, China). In addition, four types of metal sheets with a thickness of 1 mm each were selected as test materials, specifically including 85% lead–tin alloy, 90% lead–tin alloy, 95% lead–tin alloy, and 99% pure lead. These metal sheets were produced by Shandong Santai Radiation Protection Materials Co., Ltd. During the experiment, OxyGuard oxygen-permeable membranes, OxyGuard electrodes, and OxyGuard electrolytes produced by OxyGuard, Denmark, were also used, along with conventional laboratory materials such as KCl, a stopwatch, a beaker, a stirrer, anaerobic water, and ice cubes.

Table 1 lists the main equipment used in this experiment. All equipment has been tested and calibrated, with stable performance and accurate data, meeting all the requirements of the experiment.

### 2.2. Experimental Method

#### 2.2.1. Preparation of Electrolyte Solution

Initially, 14.91 g of KCl powder was weighed using a balance and poured into a 2 L beaker. Approximately 800 mL of distilled water was added, and the mixture was stirred using a magnetic stirrer until the KCl was completely dissolved. Distilled water was then added to reach a total volume of 1 L, followed by gentle stirring with a stirrer to ensure a uniform concentration. This resulted in a 1 L KCl solution with a concentration of 0.2 mol/L. To obtain KCl electrolyte solutions of different concentrations, 29.82 g, 44.73 g, 59.64 g, and 74.55 g of KCl powder were weighed separately and prepared following the aforementioned steps to yield KCl electrolyte solutions with concentrations of 0.4 mol/L, 0.6 mol/L, 0.8 mol/L, and 1.0 mol/L, respectively.

#### 2.2.2. Assembly of DO Sensor

**Figure 2:**
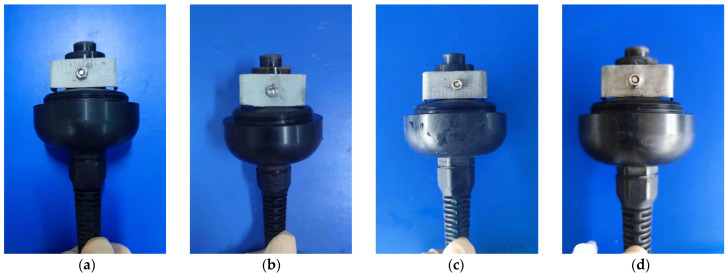
Installation of electrodes. (**a**) 85% lead–tin alloy; (**b**) 90% lead–tin alloy; (**c**) 95% lead–tin alloy; (**d**) 99% pure lead.

(1) Assembly of Sensor with Corresponding Oxygen-Permeable Membrane: Firstly, six types of oxygen-permeable membranes (0.015 mm PFA, 0.03 mm PFA, 0.05 mm PFA, 0.015 mm FEP, 0.03 mm FEP, and 0.05 mm FEP) were cut into circles with a diameter of 20 mm, matching the diameter of the OxyGuard oxygen-permeable membrane. Subsequently, the OxyGuard DO sensor was dismantled by unscrewing the nut and sensor cavity to drain the original electrolyte. After removing the original oxygen-permeable membrane and rubber ring, all components were rinsed with water, wiped clean, and dried. Next, a new rubber ring and the tested oxygen-permeable membrane were placed inside the sensor cavity, followed by tightly securing the oxygen-permeable membrane cover. OxyGuard electrolyte was injected to ensure the cavity was completely filled while removing any excess electrolyte and air. Finally, the reassembled sensor was left to stand in the air for 24 h to ensure complete polarization. Through these steps, six new DO sensors were successfully constructed, and their detailed configurations can be referred to in Table 2.

(2) Assembly of Sensor with Corresponding Electrodes and Electrolyte: In this study, four different electrode materials were utilized, including 99% pure lead and 85%, 90%, and 95% lead–tin alloys. These materials were processed into uniformly sized “U”-shaped electrodes with a drilled hole in the middle for easy installation. The OxyGuard DO sensor was then dismantled, and the original OxyGuard electrode was removed and replaced with the tested electrode (Figure 2). Five concentrations of electrolyte (0.2 mol/L, 0.4 mol/L, 0.6 mol/L, 0.8 mol/L, 1.0 mol/L) were used in combination with four different proportions of lead–tin alloy electrodes, which were installed in the OxyGuard DO sensor. The OxyGuard oxygen-permeable membrane was installed, the tested electrolyte was injected, and the sensor was tightened and allowed to fully polarize. A total of 20 galvanic DO sensors were constructed, as detailed in Table 3.

#### 2.2.3. Experiment on the Effect of Oxygen Permeability on the Output Signal of DO Sensors

In this experiment, 50 L of clean water was first injected into the test tank. Subsequently, the DO sensor was fixed at the center position 5 cm below the water surface. A heating rod and a submersible pump were used, with the former gradually increasing the water temperature and the latter ensuring a water flow speed of no less than 0.3 m/s. A multimeter was connected to the output voltage terminal of the DO sensor. For convenient temperature measurement, an NTC resistor was fixed beside the DO sensor, and another multimeter was used to measure its resistance. The relationship between the NTC resistor and temperature is shown in Equation (4). At the beginning of the experiment, the initial water temperature was recorded, and then the water temperature was gradually increased by 25 °C. During this process, the resistance value of the NTC resistor and the output voltage data of the DO sensor were continuously recorded. The above experimental steps were repeated for the six DO sensors listed in Table 2. In addition, to eliminate the influence of water quality changes on the experimental results, new test water was replaced after each test to ensure the consistency of the test conditions and the comparability of the data.

(4) $$R_{t}=R\cdot e^{\left(B\cdot \left(\frac{1}{T_{1}}-\frac{1}{T_{2}}\right)\right)}$$

In the formula: *T*_1_ and *T*_2_: Kelvin temperature, the unit is K; *R_t_:* resistance at temperature *T*_1_, the unit is Ω; *R*: nominal resistance at temperature *T*_2_ (usually referring to room temperature, such as 25 °C), the unit is Ω; *B*: temperature coefficient, and the *B* value of the NTC in the experiment is 3950.

#### 2.2.4. Experiment on the Stability of DO Sensor Output Signal Affected by Sensing Components

Twenty-six reassembled DO sensors, listed in Table 2 and Table 3, were placed in the center of a heat-insulated foam box. To ensure accurate temperature measurement, a mercury thermometer was fixed beside each sensor. Simultaneously, the signal output terminal of the DO sensor was connected to the voltage range of the multimeter to monitor and record changes in the sensor’s output voltage in real time. To investigate the stability of the output signal, ice cubes were used to gradually lower the temperature inside the heat-insulated foam box. During the experiment, the temperature was gradually reduced from 25 °C to 10 °C, and the voltage readings displayed on the multimeter were recorded at each set temperature point. These data will be used to further analyze the relationship between temperature and the output voltage of the DO sensor, thereby assessing the sensor’s stability under different temperature conditions.

#### 2.2.5. Experiment on the Response Time of DO Sensors Affected by Sensing Components

Firstly, the voltage output terminal of the DO sensor was connected to a multimeter to measure the sensor’s voltage output value in the air. This value, considered the voltage output under saturated DO conditions, was recorded as the corresponding saturated DO voltage. Next, the sensors were placed in anaerobic water, ensuring they were located 5 cm below the water surface. After the voltage stabilized, the sensor was quickly moved to the air, and a stopwatch was started to time when the sensor probe came into contact with the air. While observing the multimeter, we waited for the measured voltage to reach 90% of the corresponding saturated DO voltage value. Once this threshold was reached, the stopwatch was immediately stopped, and the displayed time was recorded as the response time for each DO sensor. The aforementioned experiments were conducted on the 26 reassembled DO sensors listed in Table 2 and Table 3, and their respective response times were obtained.

#### 2.2.6. Evaluation Indicators and Data Processing

The coefficient of variation is a dimensionless statistic used to measure the dispersion degree of data distribution. It is a relative value used to compare the dispersion degree of data sets with different means and units. A smaller coefficient of variation indicates less dispersion and better stability of the data; conversely, a larger coefficient of variation suggests greater dispersion and poorer stability. The calculation formula is as follows:

(5) $$CV=\frac{\sigma }{\mu }$$

In the formula: *CV* is the coefficient of variation, *σ* is the standard deviation, and *μ* is the mean.

The formula for calculating the oxygen permeability rate of the galvanic dissolved oxygen sensor is as follows:

(6) $$J=\frac{\Delta c\cdot v}{t\cdot s}$$

In the formula: J: diffusion rate, the unit is mol/(cm^2^⋅s); Δc: dissolved oxygen concentration change, the unit is mol/L; v: electrolyte volume, the unit is L; t: response time, the unit is s; s: area of oxygen-permeable membrane, the unit is cm^2^. In the experiment, all oxygen-permeable membranes are circular with a diameter of 2 cm, and the electrolyte volume is 18 mL for each.

In this study, data analysis was primarily performed using Excel 2020, and data fitting and graph plotting were carried out using Origin 2021.

## 3. Results and Discussion

### 3.1. The Influence of Oxygen Permeability Efficiency on the Output Signal of DO Sensors

**Figure 3:**
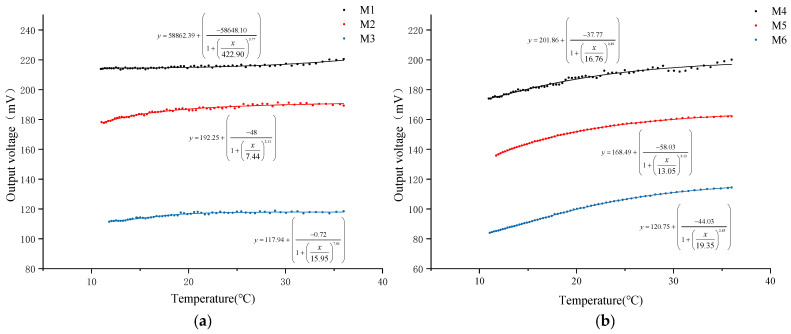
Relationship between temperature and DO sensor output voltage. (**a**) DO sensor corresponding to PFA; (**b**) DO sensor corresponding to FEP.

In this study, a water heating test was conducted on six types of DO sensors equipped with PFA and FEP membranes of three thicknesses (0.015 mm, 0.03 mm, and 0.05 mm) to systematically analyze the impact of oxygen permeability on the output signal of DO sensors. During the test, changes in NTC resistance and output voltage of each sensor were recorded as the water temperature rose. The water temperature was calculated using Formula (4), and the relationship between water temperature and the output voltage of each sensor was plotted, as shown in Figure 3.

The experimental results indicate that as the water temperature gradually increases, the output voltage of all DO sensors exhibits an upward trend. Further calculation and analysis (Table 4) reveal that when the water temperature increases by 25 °C, the output voltage increase in DO sensors with PFA membranes of 0.015 mm, 0.03 mm, and 0.05 mm thicknesses is 3.09%, 6.23%, and 6.28%, respectively. For FEP membranes of the same thicknesses, the output voltage increases are 15.00%, 19.46%, and 36.19%, respectively. The comparison data show that under the same membrane thickness, the oxygen permeability efficiency of the FEP material is more significantly affected by temperature. Simultaneously, it is observed that for membranes of the same material, the degree of temperature influence on oxygen permeability efficiency is positively correlated with the membrane thickness, meaning the thicker the membrane, the greater the temperature’s impact on oxygen permeability efficiency.

Further analysis of the data reveals a nonlinear growth trend in the relationship between the output voltage of the DO sensor and temperature. To quantify this relationship, a logistic fitting analysis was performed, and the correlation between temperature and output voltage was evaluated using the fitting correlation coefficient (*R*^2^) (Table 4). The analysis results show that under the same thickness, the correlation coefficient between the output voltage and temperature of the DO sensor corresponding to the FEP membrane is significantly higher than that of the PFA membrane. Specifically, the fitting coefficients for FEP membranes with thicknesses of 0.03 mm and 0.05 mm both exceed 0.99, indicating a very high correlation. However, the fitting coefficient for the 0.015 mm membrane is the lowest among the same material, with a value of only 0.85 for the PFA membrane. This suggests that an excessively thin membrane may reduce the correlation between sensor output signal and temperature, thereby affecting the accuracy of the DO sensor.

**Figure 4:**
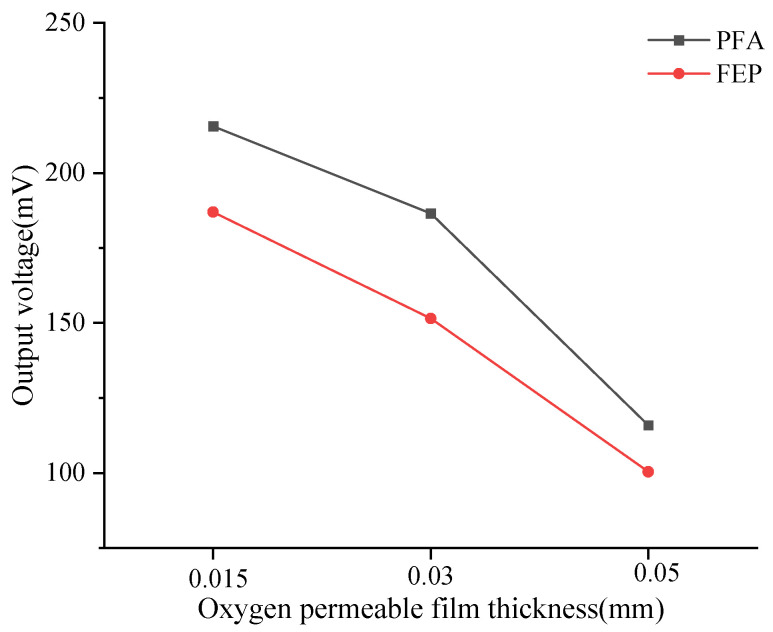
Relationship between mean output voltage and temperature of DO sensors corresponding to oxygen-permeable membranes.

In addition, this study calculated the average output voltage of the dissolved oxygen sensor corresponding to six types of oxygen-permeable membranes within the experimental temperature range (Table 4), which is visualized in Figure 4. When the membrane thickness decreased from 0.05 mm to 0.015 mm, significant changes in the output voltage of the DO sensors corresponding to PFA and FEP were observed. Specifically, the sensor output voltage corresponding to the PFA oxygen-permeable membrane increased by 86%, while the sensor output voltage corresponding to the FEP oxygen-permeable membrane increased by 74.91%. This increasing trend indicates that as the membrane thickness decreases, oxygen permeability enhances, thereby increasing the sensor’s output voltage. According to the experimental results, under the same membrane thickness, the output voltage of the PFA membrane is higher than that of FEP, which may be related to the differences in oxygen permeability between the two materials themselves.

For membranes of the same material, the thinner the membrane, the higher the output voltage, indicating superior oxygen permeability in water. For instance, under the same conditions such as temperature, when the thickness of the oxygen-permeable membrane decreases, oxygen permeability increases. This aligns with the findings of Liao et al. [28], who drew a similar conclusion through experiments. Specifically, at a temperature of 900 °C, the oxygen permeation flux of a 1.2 mm thick BBCT flake-like oxygen-permeable membrane was 1.75 mL/min.cm^2^, but when the membrane thickness was reduced to 0.8 mm, the oxygen permeation flux rapidly increased to 2.86 mL/min.cm^2^. This is mainly because a thinner oxygen-permeable membrane shortens the path for oxygen ions to pass through the bulk phase, thereby enhancing oxygen permeability. The Wagner formula [29] states that oxygen permeation flux is inversely proportional to the thickness of the oxygen-permeable membrane. Therefore, to improve oxygen permeability, efforts can be made to reduce membrane thickness.

In terms of material properties, both PFA and FEP films are melt-processable perfluoropolymers. When reacting with various monomers, these two films exhibit high radiation resistance, stabilize free radicals, and have high grafting yields [30]. These characteristics allow them to be prepared into oxygen-permeable membranes without affecting their inherent mechanical properties. Among them, PFA oxygen-permeable membranes demonstrate superior performance in terms of chemical stability, heat resistance, and other aspects, while FEP oxygen-permeable membranes are highly regarded for their efficient oxygen permeability and chemical resistance [31]. Overall, due to their unique physical and chemical properties, PFA and FEP films have broad application prospects in the field of oxygen-permeable membrane preparation.

### 3.2. The Influence of Sensing Components on the Stability of DO Sensor Output Signals

**Figure 5:**
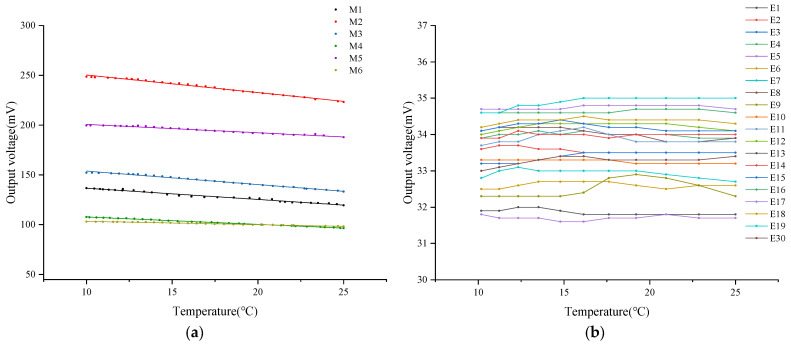
Relationship between temperature and DO sensor output voltage in air. (**a**) DO sensors with different oxygen-permeable membranes; (**b**) DO sensors with different combinations of electrodes and electrolytes.

Under conditions of constant DO concentration in the air, this study measured the output voltage of six different DO sensors equipped with tested oxygen-permeable membranes within a temperature range of 10 °C to 25 °C. Relevant data are detailed in Figure 5a. Experimental results indicate that as the ambient temperature gradually increases, the output voltage of these sensors generally exhibits a linear downward trend. Through the application of linear fitting analysis techniques, it was found that there is a significant linear correlation between the sensor’s output voltage and ambient temperature, with fitting coefficients exceeding 98%. This data are recorded in detail in Table 5. Comparative analysis shows that under the same oxygen-permeable membrane thickness conditions, the slope corresponding to PFA material is higher than that of FEP material. Therefore, the voltage drop of the former sensor is more pronounced when the temperature increases, and the mean value of its output voltage is relatively higher. This phenomenon may be attributed to the high sensitivity of PFA material to temperature changes, resulting in more oxygen molecules penetrating the membrane layer under the same temperature changes, thereby significantly affecting the voltage output.

However, unlike the output voltage of sensors in clean water, when comparing DO sensors with different thicknesses of oxygen-permeable membranes made of the same material, it was found that the 0.03 mm thick oxygen-permeable membrane exhibited the largest voltage drop among all the tested samples. Relatively speaking, the DO sensors corresponding to 0.015 mm and 0.05 mm thick oxygen-permeable membranes showed relatively similar characteristics in terms of output voltage drop. This observation may indicate that in the air, the 0.03 mm thick oxygen-permeable membrane is most sensitive to temperature changes, and its oxygen permeability is also superior. The 0.015 mm and 0.05 mm thick oxygen-permeable membranes have a relatively consistent response to temperature changes in the air.

On the other hand, this study delved into the influence of different ratios of lead–tin alloy electrodes combined with various concentrations of KCl electrolyte on the stability of the DO sensor’s output voltage. Relevant experimental results are shown in Figure 5b. Research data indicate that despite changes in air temperature, the output voltage of these sensors remains relatively stable overall. To further quantify this stability, the mean output voltage of each DO sensor was calculated, and the corresponding coefficient of variation was derived based on Equation (5). Detailed data are listed in Table 6.

Within the tested temperature range, a comprehensive analysis was conducted on the output voltage of 20 dissolved oxygen sensors. The results indicated that the mean output voltage of these sensors ranged from 31.70 mV to 34.88 mV, with a maximum *CV* of 0.71%. This peak *CV* was observed in the combination of 0.8 mol/L KCl electrolyte and 90% lead–tin alloy electrode. Compared to other combinations, which had a maximum *CV* of 0.44%, this value was slightly higher.

Although a *CV* of 0.71% is not particularly significant, it still stands out compared to other combinations. This discrepancy could arise from multiple factors, including experimental errors. In complex chemical and electrochemical processes, slight variations in experimental conditions or minor inaccuracies in measuring equipment can lead to slight fluctuations in output voltage. Additionally, this variation might reflect the sensitivity of dissolved oxygen sensors to external condition changes under specific electrolyte concentrations and electrode material ratios.

Overall, the results suggest that the proportion of lead–tin electrodes and the concentration of KCl electrolyte have minimal impact on the amplitude of the dissolved oxygen sensor’s output voltage. Furthermore, the corresponding sensors exhibit excellent stability across various temperatures. This could be due to the relatively small output signal of the galvanic dissolved oxygen sensor, resulting in less significant differences. Within the tested temperature range, the effect of temperature on the electrode reaction rate is not pronounced, or changes in reaction rates do not significantly affect the output voltage. Moreover, maintaining a constant and adequate dissolved oxygen concentration may also contribute to stabilizing the output voltage.

Figure 5a,b present differences in output voltage stability between DO sensors composed of different oxygen-permeable membranes, as well as different electrode and electrolyte combinations. These differences primarily stem from the characteristics of the oxygen-permeable membrane. The OxyGuard DO sensor has a built-in temperature compensation mechanism [32]. Therefore, even after replacing the electrode and electrolyte, it can still effectively compensate for temperature changes in the OxyGuard oxygen-permeable membrane. However, when the oxygen-permeable membrane is replaced, due to the varying permeability coefficients of different membranes, the sensor may lose its corresponding temperature compensation effectiveness.

At room temperature, temperature is the main factor affecting the permeability coefficient of oxygen-permeable membranes [33]. As the temperature of the water increases, the permeability coefficient of the oxygen-permeable membrane increases, thereby accelerating the rate of redox reactions. In a constant oxygen partial pressure environment, the output current of the DO sensor increases with increasing temperature. To eliminate the influence of temperature on the accuracy of the DO sensor, precise temperature compensation needs to be implemented based on the influence of temperature on the permeability coefficient. Since the permeability coefficient is a core parameter of the oxygen-permeable membrane, the characteristics of the oxygen-permeable membrane should be studied in detail when designing the temperature compensation circuit of the DO sensor. Vaisala, a Finnish company, achieves temperature compensation for sensors through the use of an error compensation table [34]. Similarly, Zhang et al. [35] determined the temperature coefficient K in the DO sensor through experimental research and designed a more precise temperature compensation scheme based on this. These methods achieve temperature compensation by indirectly measuring the effect of temperature on the oxygen-permeable membrane.

In this study, using saturated DO as a reference point, it was observed that six different DO sensors showed a linear downward trend with increasing temperature. This finding not only confirms that the effect of temperature on DO sensors is primarily achieved through the oxygen-permeable membrane but also reveals an approach to designing a temperature compensation mechanism for DO sensors. Specifically, in subsequent research, when designing temperature compensation schemes for different oxygen-permeable membranes, the membrane can be installed on the DO sensor, and the linear slope of its output voltage versus temperature in air can be used as an important reference indicator. Through comprehensive hardware and software corrections, it ensures that the sensor’s output signal remains stable in a saturated DO environment.

### 3.3. The Influence of Sensing Components on the Response Time of DO Sensors

Response time is a key parameter for evaluating the sensitivity and efficiency of dissolved oxygen sensors. Through in-depth research on dissolved oxygen sensors composed of different combinations of oxygen-permeable membranes, electrodes, and electrolytes, the response times of the 26 sensors listed in Table 2 and Table 3 were recorded. The oxygen permeability rates of six types of oxygen-permeable membranes were calculated according to Formula (6). Specifically, the oxygen permeability rates of PFA membranes at thicknesses of 0.015 mm, 0.03 mm, and 0.05 mm were 0.0156 mol/(cm^2^⋅s), 0.0033 mol/(cm^2^⋅s), and 0.0007 mol/(cm^2^⋅s), respectively. For FEP membranes, the corresponding oxygen permeability rates at these thicknesses were 0.0026 mol/(cm^2^⋅s), 0.0017 mol/(cm^2^⋅s), and 0.0009 mol/(cm^2^⋅s). The research results indicate a significant correlation between the thickness of the oxygen-permeable membrane and both the sensor’s response time and oxygen permeability rate. Specifically, as the thickness of the oxygen-permeable membrane increases, the sensor’s response time also shows a clear trend of prolongation.

**Figure 6:**
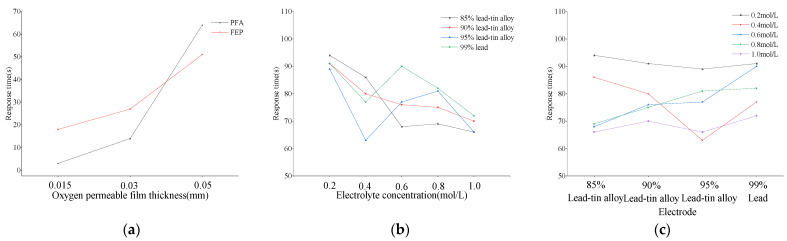
Response time of DO sensors. (**a**) DO sensors with oxygen-permeable membranes of different thicknesses; (**b**) DO sensors at different DO concentrations; (**c**) DO sensors with different electrodes.

Taking the PFA oxygen-permeable membrane as an example, when its thickness is 0.015 mm, 0.03 mm, and 0.05 mm, the corresponding response times are 3 s, 14 s, and 64 s, respectively, with an overall increase of up to 2033.33%. In comparison, the response times of the FEP oxygen-permeable membrane at the same thicknesses are 18 s, 27 s, and 51 s, respectively, with an overall increase of 183.33%. These data clearly demonstrate that both PFA and FEP oxygen-permeable membranes have a significant impact on the response time of the assembled sensor, with the thickness of the membrane playing a crucial role. It is worth noting that the influence of PFA oxygen-permeable membrane thickness on response time is more significant than that of FEP (as shown in Figure 6a).

In addition, for the 20 DO sensors assembled with electrodes of different lead–tin ratios and electrolytes of different KCl concentrations, the longest response time is 94 s, which occurs in the pairing of 0.2 mol/L KCl with 85% lead–tin alloy, while the shortest response time is 63 s, corresponding to the combination of 0.4 mol/L KCl and 95% lead–tin alloy. Further analysis (as shown in Figure 6b,c) reveals that when the electrode composition remains unchanged, the sensor’s response time slightly decreases with increasing electrolyte concentration only when the electrode is made of 85% and 90% lead–tin alloy. The response time decreases by 29.79% and 23.08%, respectively, within the concentration range from 0.2 m/L to 1.0 mol/L. Correspondingly, when the electrolyte concentration is constant, the response time increases with the increase in the lead–tin alloy ratio only at concentrations of 0.6 mol/L and 0.8 mol/L. The increase is 32.35% and 18.84%, respectively, within the ratio range from 85% to 99%. Other combinations do not show a significant upward or downward trend.

The thinner the oxygen-permeable membrane, the shorter the sensor’s response time. This is because the thin film reduces the material thickness that oxygen molecules need to penetrate, thereby accelerating the speed of oxygen molecules reaching the sensing part of the sensor [36]. As mentioned above, the thinner the membrane, the better the oxygen permeability. However, the thinner the oxygen-permeable membrane, the faster the aging effect [37]. The selection of the thickness of the oxygen-permeable membrane needs to be weighed in different application environments. For example, in polluted water, a thinner oxygen-permeable membrane may be more easily clogged by bacteria or algae, resulting in slow response. On the contrary, in harsh outdoor environments or high oxygen concentration environments, a thicker oxygen-permeable membrane may be more suitable due to its better toughness and durability, although this may come at the cost of sacrificing some rapidity.

For electrolytes and electrodes, generally speaking, the higher the electrolyte concentration, the higher the transfer efficiency between ions, thereby accelerating the speed of electrochemical reactions. This helps to shorten the sensor’s response time and improve its rapidity [38,39]. The output signal of the sensor will also be enhanced accordingly. The concentration of the electrolyte also affects the linear measurement range of the sensor. A suitable electrolyte concentration can ensure that the sensor maintains a linear output over a wide range of DO concentrations. The standard electrode potentials of lead and tin are similar, so the two metals are easily deposited, improving the overall hardness of the electrode while enhancing chemical reactions. A suitable lead–tin ratio can improve the conductivity of the electrode, thereby accelerating the speed of electrochemical reactions and shortening the response time of the DO sensor [40,41]. In this study, the influence of electrolyte concentration and lead–tin ratio on response time is relatively small compared to the oxygen-permeable membrane. Therefore, if the rapidity of the galvanic DO sensor is to be improved, the choice of oxygen-permeable membrane material and thickness is particularly important.

In summary, the material and thickness of the oxygen-permeable membrane are key factors affecting the response time of DO sensors, especially for PFA oxygen-permeable membranes, where changes in thickness have a more prominent impact on response time compared to FEP. Although electrode material and electrolyte concentration also affect the sensor’s response time to some extent, this impact is not significant relative to the oxygen-permeable membrane.

### 3.4. Performance Comparison

In this study, 0.03 mm FEP, 1.0 mol/L KCl electrolyte, and 85% lead–tin alloy electrodes were selected. These materials have demonstrated excellent performance in various previously conducted performance tests. Utilizing these optimized components, a novel DO sensor was constructed. To comprehensively evaluate the performance characteristics of this sensor, a systematic comparison test was conducted with the OxyGuard DO sensor.

During the testing process, the output terminals of the two pre-polarized sensors were first connected to a multimeter, and their saturated DO voltage values in the air were recorded. Subsequently, the sensors were smoothly transferred from the air environment to a depth of 5 cm underwater in an anaerobic environment, and timing was initiated from the instant the sensors came into contact with the anaerobic water. After the output voltage of the two DO sensors stabilized, the sensors were synchronously transferred back into the air, and their voltage changes were carefully observed and recorded.

**Figure 7:**
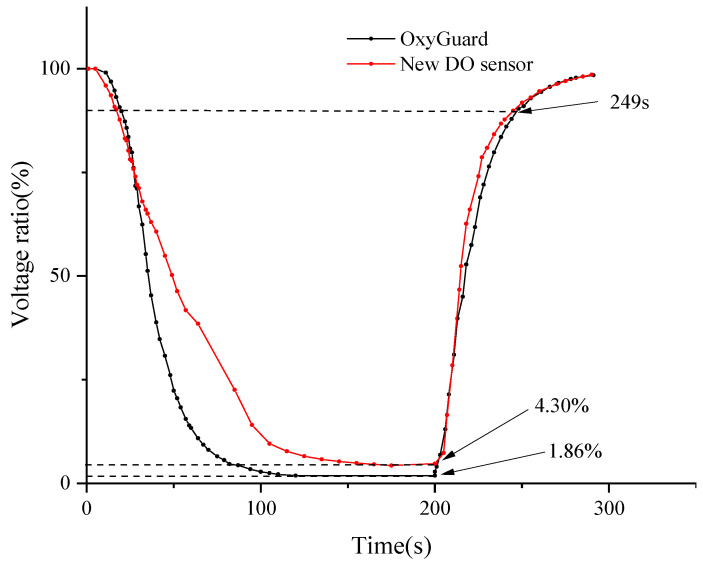
Measurement values of OxyGuard DO sensor and new DO sensor.

In DO measurement technology, the saturated DO voltage value serves as an important reference benchmark [42]. Therefore, in this study, the ratio of the output voltage to the saturated DO voltage was used as a quantitative indicator of the test results. Some of the output voltage data have been summarized in Table 7. Meanwhile, Figure 7 dynamically illustrates the trend of the ratio of output voltage to the output voltage under saturated DO conditions over time.

After in-depth data analysis of Figure 7, it was observed that during the transition from a saturated oxygen to an anaerobic environment, the OxyGuard DO sensor exhibited a faster response speed in terms of voltage stabilization. By the 200 s mark of the experiment, the output voltages of both sensors had stabilized. At this point, the measurement results of the novel DO sensor were 2.44% higher than those of the OxyGuard sensor. Subsequently, in a test where both sensors were simultaneously transferred from anaerobic water to the air, it was found that they both recovered to 90% of their respective saturated DO output voltage values within 49 s. These data indicate that the novel sensor also demonstrates impressive response speed. Nonetheless, upon comparing Figure 7, it is evident that the OxyGuard DO sensor exhibits a higher level of signal stability and accuracy. In summary, while the novel sensor performs well overall, there is still room for improvement in terms of signal stability and accuracy.

## 4. Conclusions

This study comprehensively explored the effects of oxygen-permeable membrane materials (PFA and FEP), thicknesses (0.015 mm, 0.03 mm, 0.05 mm), electrolyte concentrations (five different concentrations of KCl), and electrode ratios (four types of lead–tin alloy electrodes) on the performance of dissolved oxygen sensors, leading to a series of important conclusions. The research findings indicate that, with the same thickness, PFA oxygen-permeable membranes provide a higher output voltage compared to FEP, and their oxygen permeability is more stable, being insensitive to temperature changes. The thickness of the oxygen-permeable membrane significantly affects sensor performance, with thinner membranes exhibiting superior oxygen permeability, although excessive thinness may compromise measurement accuracy. Additionally, a linear inverse relationship between sensor output voltage and temperature was discovered, offering a new approach for temperature compensation design. The electrode ratio and electrolyte concentration have a lesser impact on the output voltage but remain non-negligible factors in optimization design. The thickness of the oxygen-permeable membrane is directly proportional to the response time, particularly for PFA membranes. Finally, the practicality of the research findings was validated through the construction and testing of a novel dissolved oxygen sensor.

## Figures and Tables

**Table 1:** Main equipment.

Equipment	Model	Production Company
NTC sensor	B3950	Shenzhen Weixin Technology Co., Ltd., located in Shenzhen, China.
DO sensor	OxyGuard Pond Master	Denmark OxyGuard Co., Ltd., located in Copenhagen, Denmark.
Magnetic stirrer	C-MAG HS7	IKA Works GmbH & Co. KG, located in Staufen, Germany.
Scale	YP10002B	Shanghai Lichen Instrument Technology Co., Ltd., located in Shanghai, China.
Insulating foam box	300 × 205 × 255	-
Water tank	755 × 530 × 500	Tengyi Plastics Co., Ltd., located in Dongguan, China.
Cylindrical bucket	30 L	Liangbao Plastics Co., Ltd., located in Wuxi, China.
Heating rod	2200 W	Xinshaoguang Electric Appliance Co., Ltd., located in Changsha, China.
Multimeter	VC890D	Victor Instrument Co., Ltd., located in Xi’an, China.

**Table 2:** Sensor configurations corresponding to oxygen-permeable membranes.

Sensor Number	Core Sensing Components		
	Oxygen-Permeable Membrane	Electrode	Electrolyte
M1	0.015 mm PFA	OxyGuard Electrode	OxyGuard Electrolyte
M2	0.03 mm PFA		
M3	0.05 mm PFA		
M4	0.015 mm FEP		
M5	0.03 mm FEP		
M6	0.05 mm FEP		

**Table 3:** DO sensor configurations corresponding to electrodes and electrolytes.

Sensor Number	Core Sensing Components			Sensor Number	Core Sensing Components		
	Oxygen-Permeable Membrane	Electrode	Electrolyte		Oxygen-Permeable Membrane	Electrode	Electrolyte
E1	OxyGuard Oxygen-Permeable Membrane	85% lead–tin alloy	KCl (0.2 mol/L)	E11	OxyGuard Oxygen-Permeable Membrane	95% lead–tin alloy	KCl (0.2 mol/L)
E2			KCl (0.4 mol/L)	E12			KCl (0.4 mol/L)
E3			KCl (0.6 mol/L)	E13			KCl (0.6 mol/L)
E4			KCl (0.8 mol/L)	E14			KCl (0.8 mol/L)
E5			KCl (1.0 mol/L)	E15			KCl (1.0 mol/L)
E6		90% lead–tin alloy	KCl (0.2 mol/L)	E16		99% pure lead	KCl (0.2 mol/L)
E7			KCl (0.4 mol/L)	E17			KCl (0.4 mol/L)
E8			KCl (0.6 mol/L)	E18			KCl (0.6 mol/L)
E9			KCl (0.8 mol/L)	E19			KCl (0.8 mol/L)
E10			KCl (1.0 mol/L)	E20			KCl (1.0 mol/L)

**Table 4:** Output voltage parameters of oxygen permeability test.

Sensor Number	Output Voltage Parameters				
	Mean Value (mV)	Initial Output Voltage (mV)	Final Output Voltage (mV)	Increase Amplitude	*R* ^2^
M1	215.55	213.8	220.4	3.09%	0.85
M2	186.49	178.1	189.2	6.23%	0.96
M3	115.92	111.4	118.4	6.28%	0.95
M4	187.00	174.0	200.1	15.00%	0.97
M5	151.50	135.7	162.1	19.46%	0.99
M6	100.40	84.0	114.4	36.19%	0.99

**Table 5:** Linear fitting parameters between sensor output voltage and temperature.

Sensor Number	Output Voltage Parameters			
	Mean Value (mV)	Slope	Intercept	*R* ^2^
M1	130.84	−1.15	148.23	0.98
M2	237.67	−1.90	270.70	0.99
M3	142.93	−1.44	169.13	0.99
M4	101.21	−0.74	115.21	0.99
M5	194.73	−0.89	210.26	0.98
M6	101.13	−0.36	107.27	0.99

**Table 6:** Fluctuation-related parameters of DO sensor output voltage in air.

Sensor Number	Output Voltage Parameters			Sensor Number	Output Voltage Parameters		
	Mean Value (mV)	Standard Deviation	Coefficient of Variation (%)		Mean Value (mV)	Standard Deviation	Coefficient of Variation (%)
E1	31.86	0.07	0.24	E11	33.89	0.15	0.44
E2	33.56	0.07	0.22	E12	34.21	0.10	0.30
E3	33.39	0.13	0.39	E13	34.37	0.07	0.21
E4	33.99	0.06	0.19	E14	33.98	0.05	0.10
E5	34.74	0.04	0.14	E15	34.20	0.09	0.29
E6	32.60	0.07	0.24	E16	34.62	0.04	0.12
E7	32.93	0.11	0.34	E17	31.70	0.06	0.19
E8	34.04	0.14	0.44	E18	34.37	0.07	0.21
E9	32.48	0.23	0.71	E19	34.88	0.15	0.43
E10	33.26	0.04	0.14	E20	33.27	0.12	0.36

**Table 7:** Measurement values of OxyGuard DO sensor and new DO sensor.

Status	Time (s)	Output Voltage (mV)	
		OxyGuard DO Sensor	New DO Sensor
Saturation oxygen	-	32.2	174.6
Decrease to anaerobic	19	29.2	153.1
	40	12.5	106.0
	57	5.0	72.9
	85	1.4	39.4
	105	0.8	16.7
Anaerobic	-	0.6	7.5
Increase to saturation oxygen	1	0.9	8.3
	19	17.0	109.3
	39	26.9	151.5
	49	29.1	159.5

## Data Availability

The data presented in this study are available on request from the corresponding author.
